# Neuroimaging assessment in Down syndrome: a pictorial review

**DOI:** 10.1186/s13244-019-0729-3

**Published:** 2019-05-20

**Authors:** Marta Rodrigues, Joana Nunes, Sofia Figueiredo, António Martins de Campos, Ana Filipa Geraldo

**Affiliations:** 1 Neuroradiology Department, Centro Hospitalar de Vila Nova de Gaia/Espinho, R. Conceição Fernandes, 1079 Vila Nova de Gaia, Portugal; 2 Neurology Department, Centro Hospitalar de Vila Nova de Gaia/Espinho, Vila Nova de Gaia, Portugal

**Keywords:** Down syndrome, Brain abnormalities, Head and neck malformations, Spine malformations, Vascular abnormalities

## Abstract

Down syndrome (DS), or trisomy 21, is the leading genetic cause of intellectual incapacity worldwide, with a reported incidence of about 1 in 1,000 to 1 in 1,100 live births. Besides the several commonly known physical features characteristic of this syndrome present at birth, DS may additionally affect every organ system. In addition, despite the large number of published papers concerning this syndrome, there is scarce literature focusing specifically in the typical neuroimaging features associated with this condition. The aim of this paper is to review and systematize the distinctive characteristics and abnormalities of the central nervous system, head and neck, and spine present in DS patients that should actively be searched for and evaluated by radiologists and/or neuroradiologists.

## Key points

- Down syndrome (DS) is the leading genetic cause of intellectual incapacity worldwide.
- There is scarce literature focusing specifically in the typical features of DS involving the central nervous system, spine, and head and neck region.
- The aim of this paper was to review and systematize the neuroimaging findings in DS patients.

## Introduction

Down syndrome (DS), or trisomy 21, is the foremost genetic cause of intellectual incapacity worldwide. The World Health Organization estimates a DS incidence of about 1 in 1,000 to 1 in 1,100 live births. During the last 20 years, there has been an increase of about 10% in the number of pregnancies with DS in Europe, which may be related to the increasing maternal age at conception [1]. It is well established that the risk for having DS affected baby increases with maternal age, with the chance of 1 in 69 for a woman with 40 years old at the time of delivery [2]. Nevertheless, the live birth prevalence remains stable, mainly due to the improvement and widespread availability of prenatal screening [1]. The prenatal screening of DS encompasses non-invasive methods that estimate the risk of having a child with DS, whereas definite diagnosis is made through genetic mapping of fetal cells [1]. The methods used for fetal screening are maternal age assessment, imaging markers in the first- and/or second-trimester ultrasounds, maternal serum biochemical testing, and, more recently, analysis of cell-free fetal DNA from maternal plasma [1].

There are several physical features characteristic of this syndrome present at birth, such as a brachycephalic shape of the head, an epicanthic fold, small and flat nose bridge, clinodactyly, single palmar crease, and augmented nuchal skin [3, 4]. DS syndrome is also commonly associated with impairments in language [5], cognition [6], learning skills, and memory [7]. Additionally, DS may affect every organ system, including the central nervous system (CNS), the head and neck region, and the vertebral column.

Recent medical advances have considerably enlarged the life span of people with DS, which is greater than 55 years, in economically developed countries [8]. Because of this increasingly better life expectancy, the number of imaging studies performed in DS patients is noticeably increasing, including neuroimaging studies. Although there are several published papers in the literature concerning DS [3, 9–11], there are few focusing specifically in the typical neuroimaging findings of this common syndrome. In this paper, we review several characteristic features and malformations present in DS patients that should be carefully evaluated in neuroimaging studies (Table 1).

**Table 1:** Major neuroimaging findings in Down syndrome patients

Organ system	Major imaging findings
Head and neck	Reduction in head size/microcephaly Brachycephaly without craniosynostosis Platybasia Macroglossia
	Stenotic external auditory canal Ossicular chain abnormalities Hypoplasia/aplasia of the bony island of the lateral semi-circular canal Semi-circular canal dehiscence Stenosis of the cochlear nerve canal Stenosis of the internal auditory canal
Brain	Overall reduced brain volume Progressive brain atrophy Basal ganglia calcifications Malformations of the corpus callosum Stroke or hemorrhage (complications of moyamoya syndrome)
Spine	Craniocervical instability Flattened surface of the occipital condyles Bifid anterior or posterior C1 arches Atlanto-occipital assimilation Congenital *Os Odontoideum* “Mickey Mouse” pelvis
Vascular	Moyamoya syndrome Aberrant subclavian artery

## Head and neck

### Brachycephaly and other typical craniofacial features

**Fig. 1:**
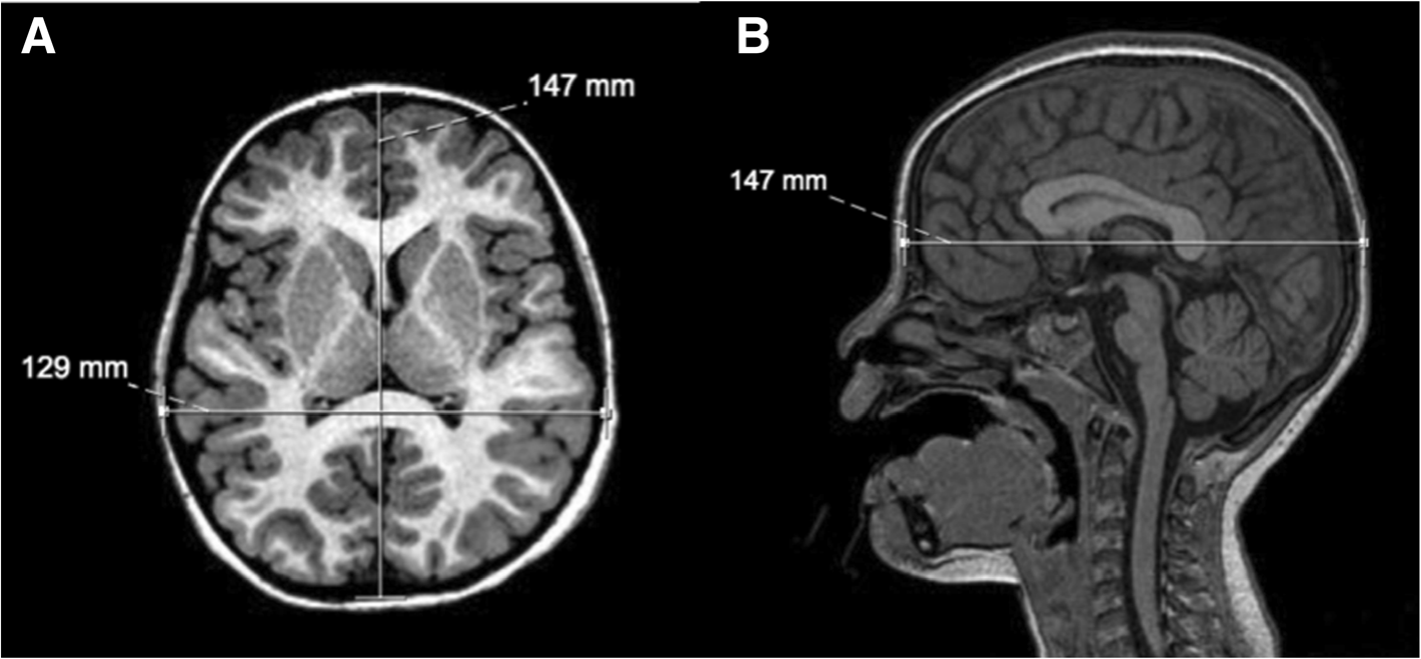
Axial (**a**) and sagittal (**b**) T1/3D in a 5-year-old child with Down syndrome. Measurements of biparietal and occipitofrontal diameters are presented, showing increased cephalic index (129/147 × 100 = 88) (normal value ranges from 74 to 83), in relation to brachycephaly, a common feature of this condition. The cranial sutures remain patent

**Fig. 2:**
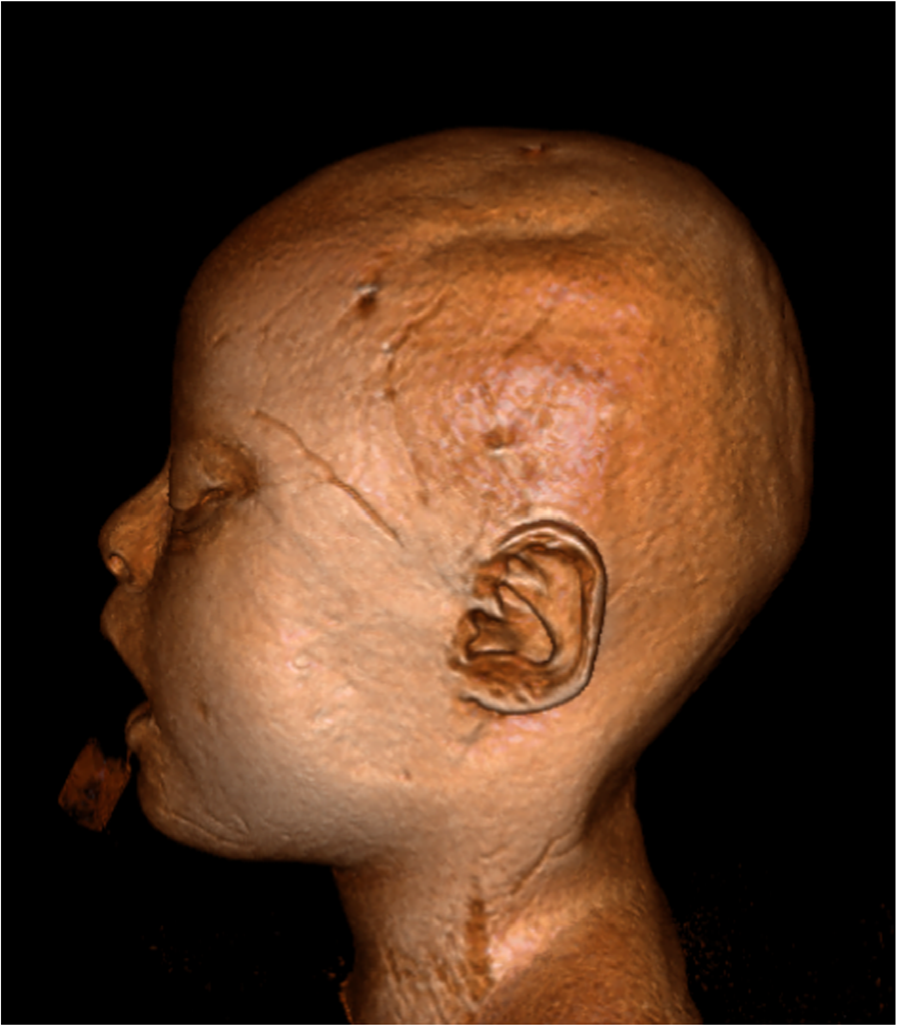
3D Reconstruction of MR T1/3D in a 5-year-old child with Down syndrome, showing flat occiput with brachycephaly. Note also the small ear with an overfolded helix, which is a common feature of Down syndrome patients

DS patients usually show a characteristic craniofacial phenotype, with multiple anomalies of the craniofacial skeleton reported in the literature. These include more often reduction in head size (microcephaly) associated with an abnormal calvarial widening in the transverse diameter, a finding known as brachycephaly [12, 13]. Therefore, in brachycephaly, the biparietal diameter (BPD) to occipitofrontal diameter (OFD) ratio (corresponding to the cephalic index) is increased and approaches the 95th percentile (cephalic index = BPD/OFD × 100). This feature can be identified visually and may also be evaluated in neuroimaging studies (Figs. 1 and 2). Although brachycephaly is frequently caused by craniosynostosis involving the coronal and lambdoid sutures limiting anteroposterior growth of the skull, in DS, the sutures typically remain patent. Besides DS, brachycephaly can be also associated with many other genetic syndromes, including Apert, Carpenter, Larsen, and Roberts syndromes [14].

Some other recognized characteristics of DS craniofacial morphology are midface hypoplasia, malocclusion with posterior cross-bite and anterior open bite, and advanced position of the tongue and macroglossia [12, 15]. Some of these features, namely the ones involving the superior airway, can increase the risk of obstructive sleep apnea in children with DS, which affects about 30 to 75% of DS individuals [3].

**Fig. 3:**
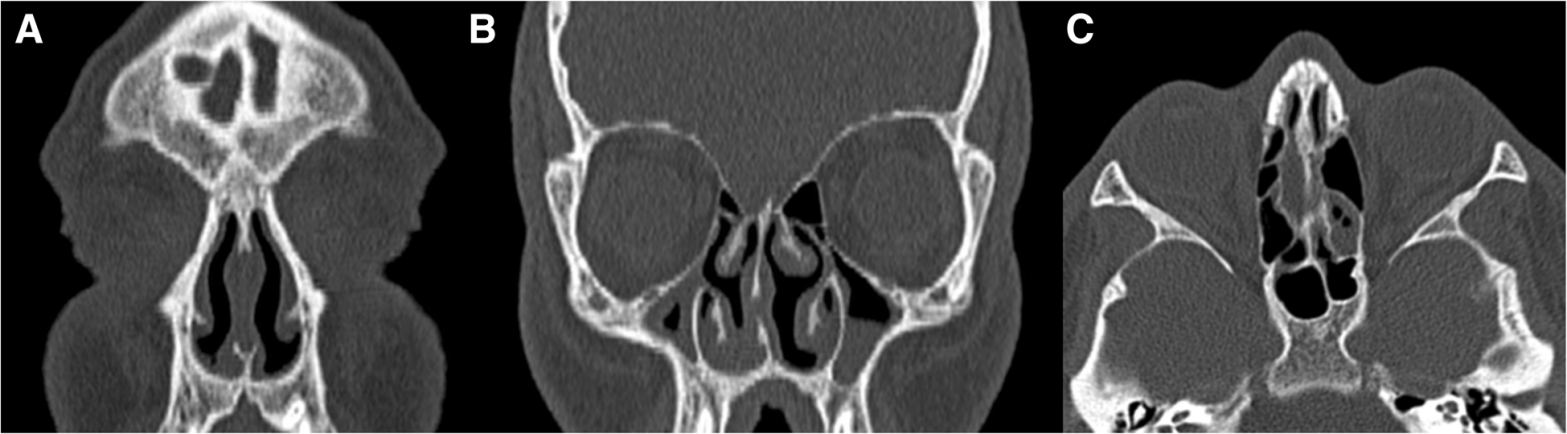
Coronal (**a**, **b**) and axial (**c**) reconstruction of paranasal sinus CT of a 21-year-old Down syndrome patient, depicting absent frontal sinuses and hypoplasia of the maxillary and sphenoid sinuses

**Fig. 4:**
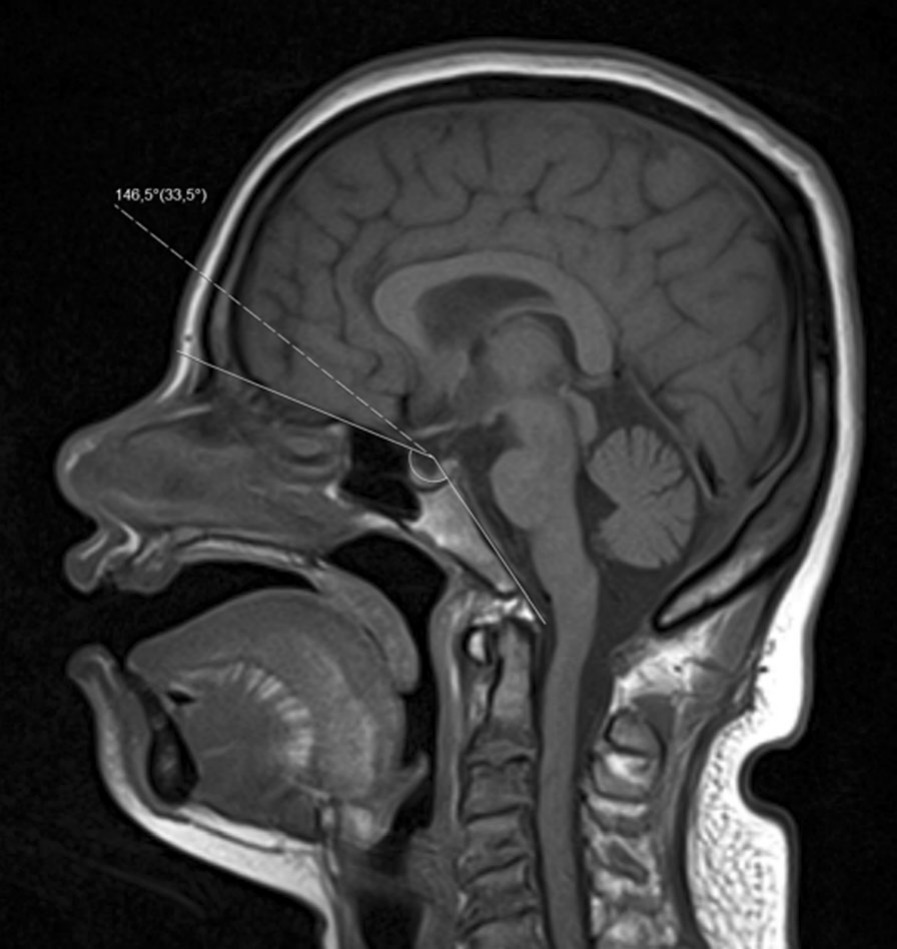
Sagittal T1/SE of a 38-year-old Down syndrome patient with platybasia (skull base angle > 143°). As shown in the figure, this angle is measured using a line joining the nasion with the center of the pituitary fossa and a line joining the anterior border of the foramen magnum and the center of the pituitary fossa

Finally, DS is associated with flattening of the skull base or platybasia, which is reflected by an increase of the skull base angle (Figs. 3 and 4). This angle is measured using a line joining the nasion with the center of the pituitary fossa and a line joining the anterior border of the foramen magnum and the center of the pituitary fossa; an angle superior to 143° defines platybasia.

### Ear abnormalities

**Fig. 5:**
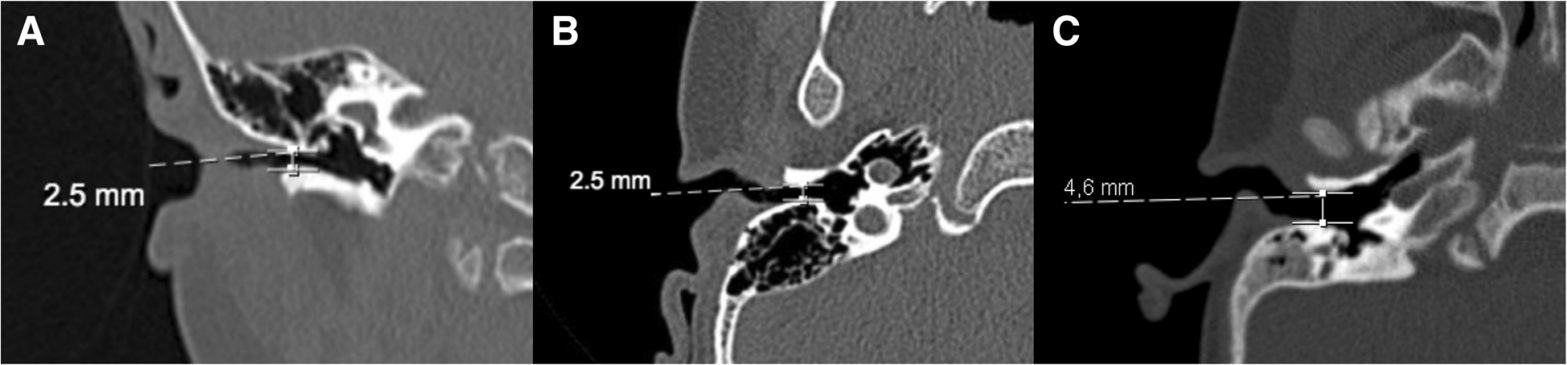
Coronal (**a**) and axial (**b**) reconstruction of a temporal bone CT of a 2-year-old boy with Down syndrome, at the level of the mesotympanum, depicting a right stenotic external auditory canal (less than 4 mm in diameter as defined by Cole and Jahrsdoerfer, 2009). Axial reconstruction (**c**) of a temporal bone CT of a normal 2-year-old boy for comparison. Measurement is done in the bony component of the external auditory canal at the most stenotic point

**Fig. 6:**
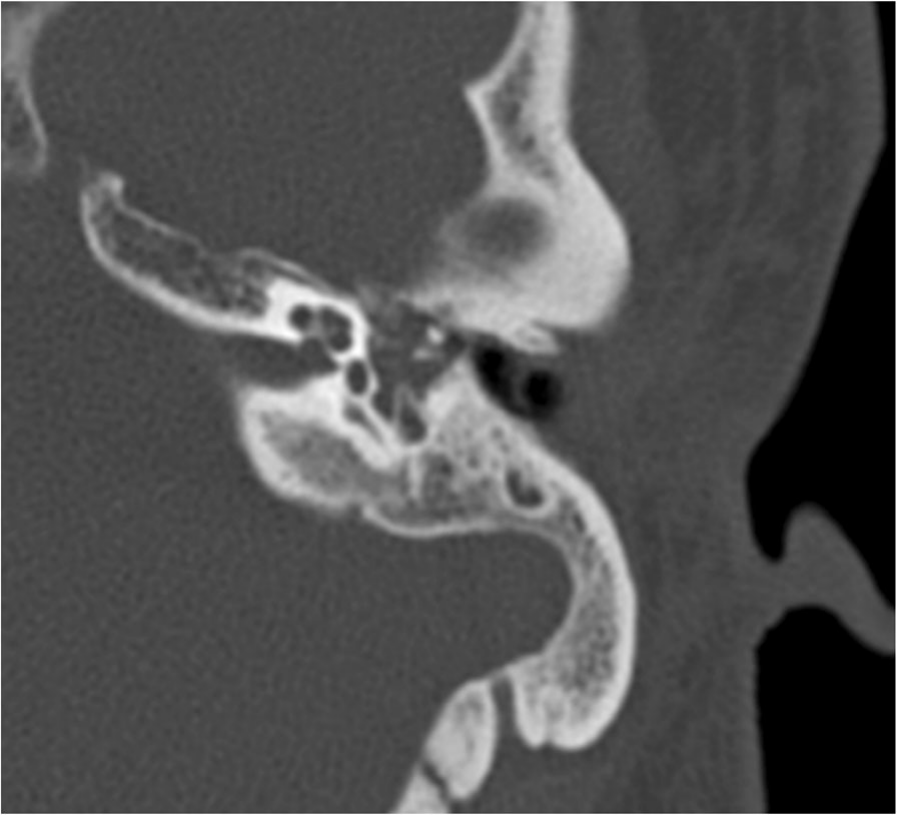
Axial reconstruction of a temporal bone CT, at the level of the ossicular chain, of a 36-year-old Down syndrome patient demonstrating hypopneumatization of the mastoid and signs of left middle chronic otitis, with an erosion of the ossicular chain, namely the stapes

Overall, DS patients have a high prevalence of hearing loss, reaching 38 to 78% of cases [16, 17]. Conductive hearing loss (CHL) is the most common subtype and usually results from sequelae of chronic otitis media (which has a higher incidence in DS). Alternatively, CHL in this population can be secondary to stenotic external auditory canal [18] (Fig. 5), dehiscence of the facial nerve canal, or ossicular chain abnormalities [19, 20]. The latter can either be related to chronic infections [17] (including erosion of the long process of the incus, erosion of the manubrium of the malleus, and erosion of the stapes) or attributed to congenital deformities, such as malformation of the stapes (Fig. 6). These findings should be considered and actively searched for with appropriate neuroimaging evaluation in DS children who have a persistent CHL despite maximal management of middle ear infections.

**Fig. 7:**
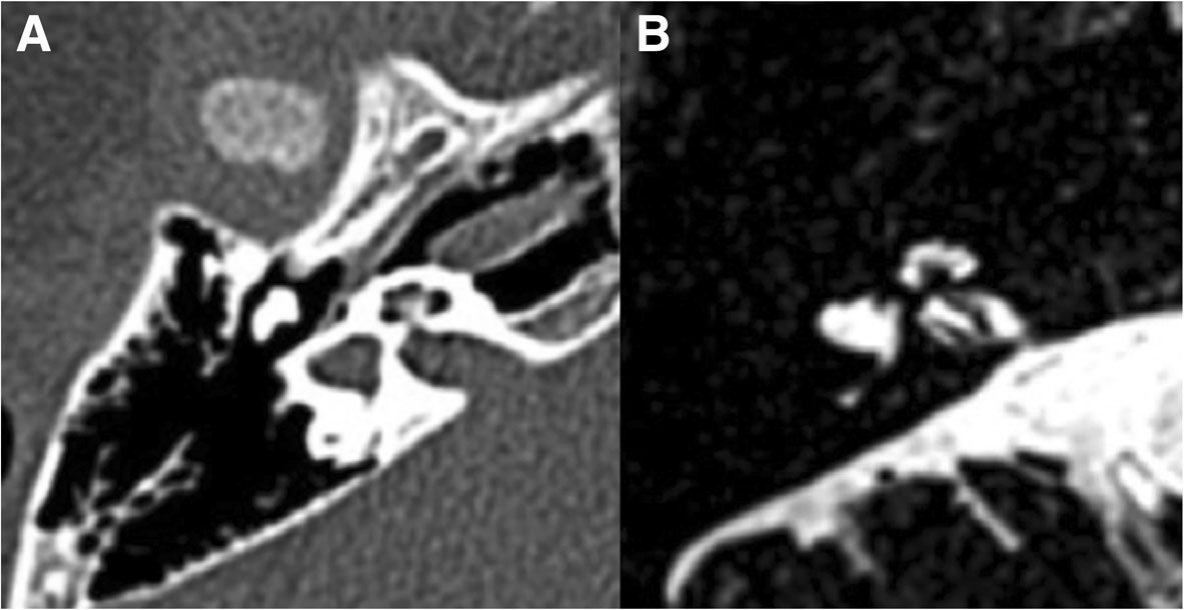
Temporal bone CT (**a**) and MRI (axial T2/3D) (**b**) from a 2-year-old patient with Down syndrome at the level of the internal auditory canal, showing complete absence of the right bone island and consequent aplasia of the lateral semi-circular canal. Note also the dysplastic vestibule as a result of the absence of the lateral semi-circular canal

**Fig. 8:**
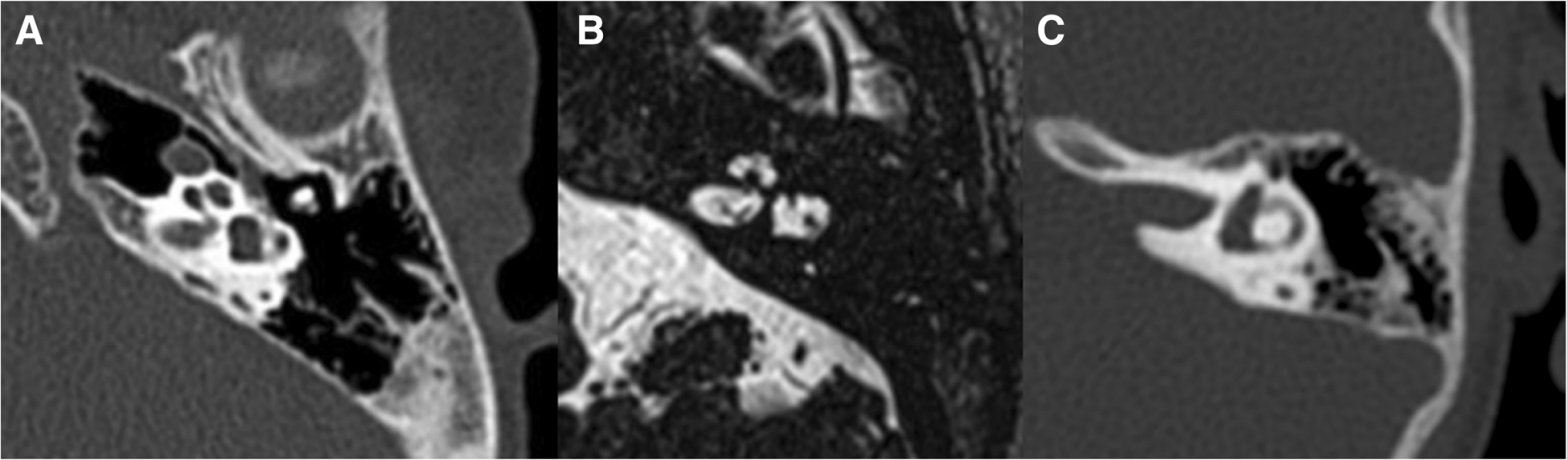
Temporal bone CT (**a**) and MRI (axial T2/3D) (**b**) from a 2-year-old Down syndrome patient at the level of the lateral semi-circular canal, revealing left hypoplastic bone island (less than 3 mm in greatest diameter). Axial temporal bone CT (**c**) at the same level of a normal 2-year-old boy for comparison

Sensorineural hearing loss (SNHL) may also occur in children with DS and results from inner ear malformations in 75% of cases [17], which can usually be readily assessed on computed tomography (CT) and/or magnetic resonance imaging (MRI) studies of the temporal bone. The most common inner ear anomaly in DS is of the bony island of the lateral semi-circular canal (LSCC), defined by a measurement of 3 mm or less, with a prevalence of about 50% [3, 17] (Fig. 7**)**. The bone island can also be totally absent, giving a shape of a small bud resembling the vestibular anlage during embryological development (Fig. 8). The absence and small size of the bone island constitute a spectrum of LSCC dysplasia. The LSCC dysplasia is more common than that of the superior or posterior semicircular canals, which is thought to result from the late formation in embryogenesis of the LSCC. Another semicircular canal anomaly frequently associated with DS is the semicircular canal dehiscence, with a reported prevalence of approximately 9%.

In addition, stenosis of the cochlear nerve canal (< 1.4 mm) and/or tight internal auditory canal (IAC) (< 3.3 mm) are present in about 20 to 25% of patients with DS and can be associated with aplasia or hypoplasia of the cochlear nerve [3, 17]. The proposed mechanism of origin of this malformation is failure of neural induction by chemotaxis, i.e., the malformation of the membranous labyrinth in patients with SNHL might inhibit the normal production of nerve growth factor, which is crucial for the normal growth of the vestibulocochlear nerve, resulting in aplasia or hypoplasia of the cochlear nerve. Also, the normal development of the IAC requires a stimulus from the cochlear nerve, leading, in its absence, to an arrest of IAC formation. Several other cochlear anomalies have also been described in DS patients, such as a short cochlea as well as the Mondini anomaly, represented by an anomalous cochlea (with 1.5 turns, instead of the usual 2.5 turns), enlarged vestibule with normal semi-circular canals, and enlarged vestibular aqueduct [17].

Routine screening for hearing loss in DS patients has been recommended, as early identification and treatment might prevent long-term sequelae and minimize impairment in life quality [21]. Indeed, amplification with hearing aids, or with cochlear implants, can improve the communication skills of DS patients with SNHL [17].

## Brain

### Reduced brain volume when compared with normal age-matched individuals

**Fig. 9:**
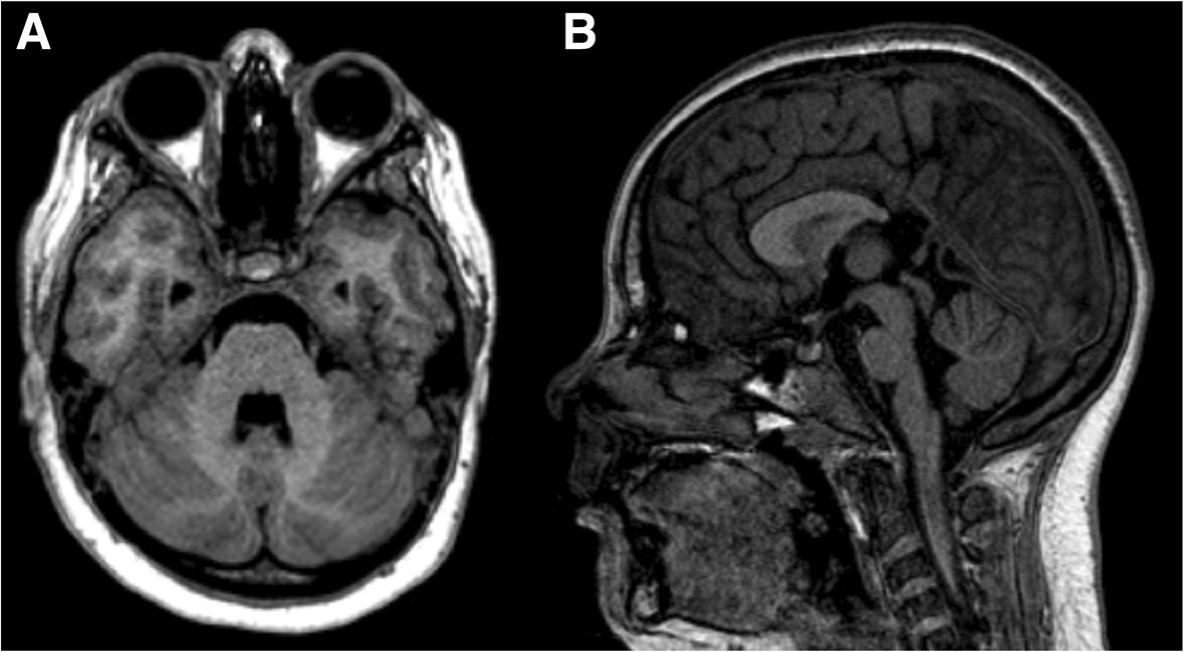
Axial (**a**) and sagittal (**b**) T1/3D of a 12-year-old girl with Down syndrome, showing small brainstem structures with enlargement of the IV ventricle. In particular, there is a reduced cranio-caudal diameter of the pons. Also, the agenesis of the splenium of corpus callosum, a finding that has been occasionally described in Down syndrome

Brain MRI assessment in DS patients of different ages has showed globally reduced total brain volume in this subgroup of patients when compared with age-matched controls [22], with a difference of about 20% less in total volume; the reduction in brain size appears in 4–5-month fetuses and becomes more evident during the last trimester [23] and postnatally [22]. The major changes are reported in the brainstem (specially the pons), hippocampi, and frontal lobes [22]. Another common finding of this syndrome is cerebellar and vermian hypoplasia [11]. Interestingly, the deep gray matter structures maintain normal in volume [11, 24] (Fig. 9).

### Progressive brain atrophy and related disorders

Besides the overall reduced total brain volume, evidence also suggests that DS patients experience premature brain aging, with accelerated volume loss [25]. The incidence of age-related cognitive decline and dementia is therefore greater in patients with DS compared to the general population and also develop sooner in life [26]. The prevalence of dementia in DS patients can be almost 55% at 60 years of age [27].

For DS individuals older than 40 years, dementia occurs in a similar pattern to Alzheimer disease (AD), with a decline in memory and impairment of language function [26]. In fact, it is reported that older DS patients show neuropathological changes characteristic of AD, namely increased cerebral beta-amyloid deposition, neurofibrillary tau tangles, neuritic plaques, and neuron cell loss [25].

**Fig. 10:**
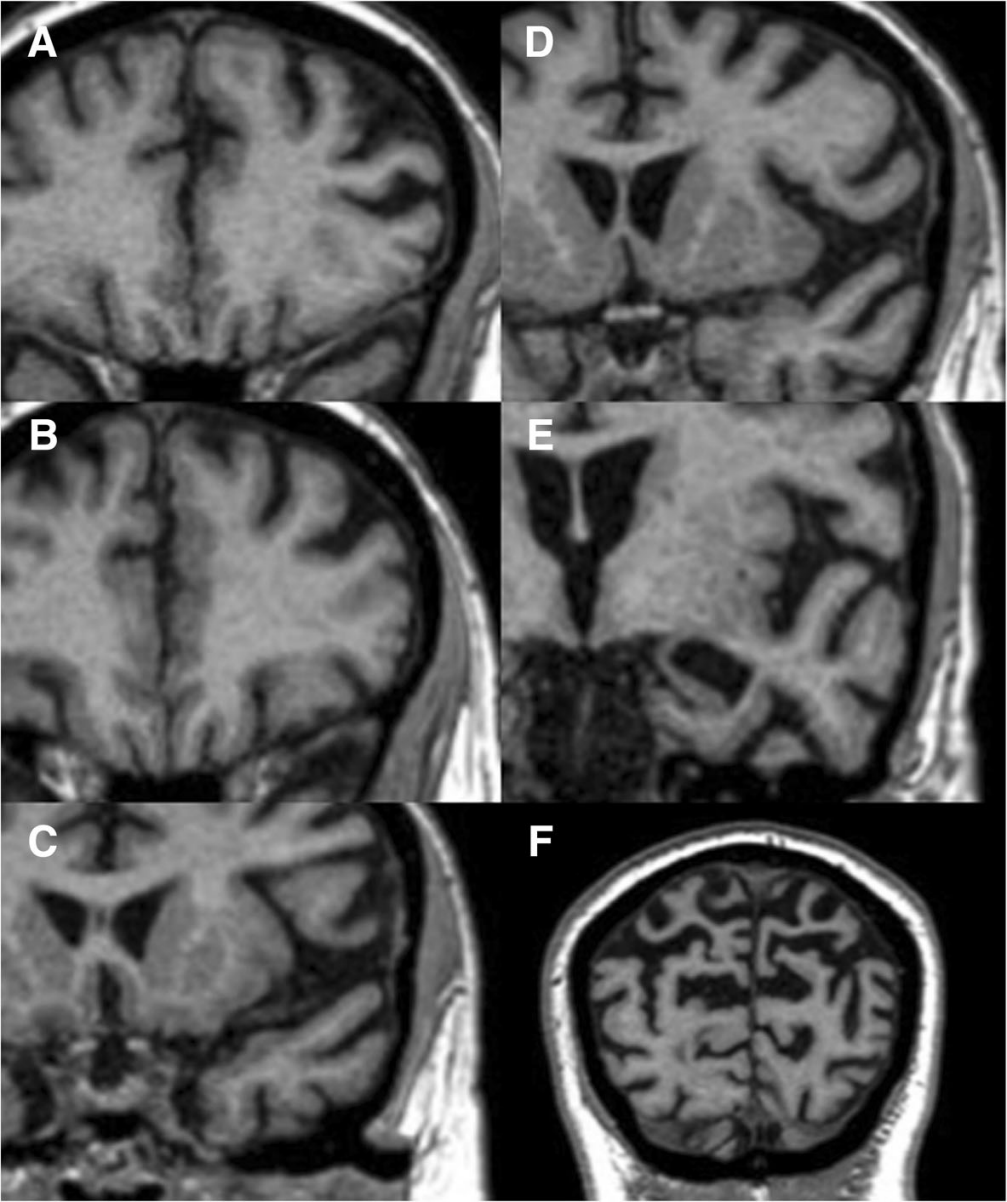
Coronal T1/3D reconstructions of a 48-year-old Down syndrome patient with dementia. **a** Grade 2 anterior cingulate gyrus atrophy (MRI visual rating scale). **b** Grade 2 orbito-frontal cortex volume loss. **c** Grade 2 anterior temporal cortex atrophy. **d** Grade 3 fronto-insular loss of volume. **e** Grade 4 medial temporal atrophy, including the amygdala and hippocampus. **f** Grade 3 posterior parieto-occipital cortex atrophy

As a consequence of these similar clinical and structural changes, DS patients with dementia have MRI findings that are comparable to AD patients [3, 28], which includes diffuse cortical atrophy mainly in the parietal lobes, usually symmetric with an anterior-to-posterior grading and also broadening of the marginal branch of cingulate sulcus, central, post-central, intraparietal, and parieto-occipital sulcus; the entorhinal cortex, the amygdala, and the hippocampus are usually also involved [26, 27] (Fig. 10). Some of these findings, particularly the hippocampal volume loss, were also reported in DS patients without definitively established dementia [25, 28]. Therefore, brain MRI might assist in the early diagnosis of dementia.

**Fig. 11:**
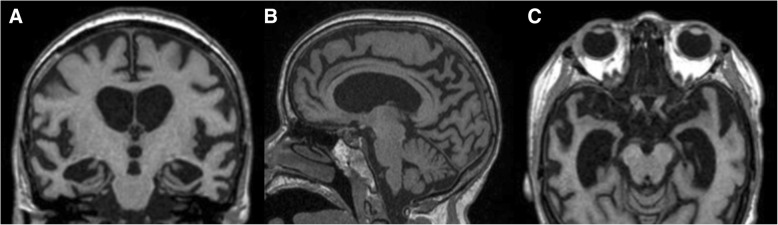
Coronal (**a**), sagittal (**b**), and axial (**c**) T1/3D reconstructions of a 58-year-old Down syndrome patient presenting with new-onset myoclonic seizures and showing imaging findings similar to Alzheimer disease, with diffuse atrophy predominantly involving the temporal mesial (**a**, **c**) (grade 4 in the visual rating scale) and parietal structures with ex-vacuo widening of adjacent CSF spaces (**b**). The clinical and radiological picture allowed the diagnosis of late-onset myoclonic epilepsy in Down syndrome (LOMEDS)

In addition, a subgroup of DS patients can develop, besides cognitive decline, a rarely described clinical and radiologic entity known as late-onset myoclonic epilepsy in Down syndrome (LOMEDS). LOMEDS is characterized by myoclonus, generalized myoclonic-tonic, and tonic-clonic seizures appearing in adult DS patients [29, 30] and is thought to result from the combination of a DS background with structural changes related to AD [31, 32] (Fig. 11). Acknowledgment and diagnosis of this syndrome is critical since it has prognostic implications and entails proper management (such as treatment with levetiracetam) [33]. The medical advances and the improved life-span of DS patients might expose more LOMEDS cases, enabling further understanding of its etiopathologic mechanisms.

Besides the typical seizures seen in LOMEDS, other forms of epilepsy are also common in DS patients, with an overall prevalence that can reach up to 46% in patients with more than 50 years old, causing significant morbidity [33].

### Basal ganglia calcifications

**Fig. 12:**
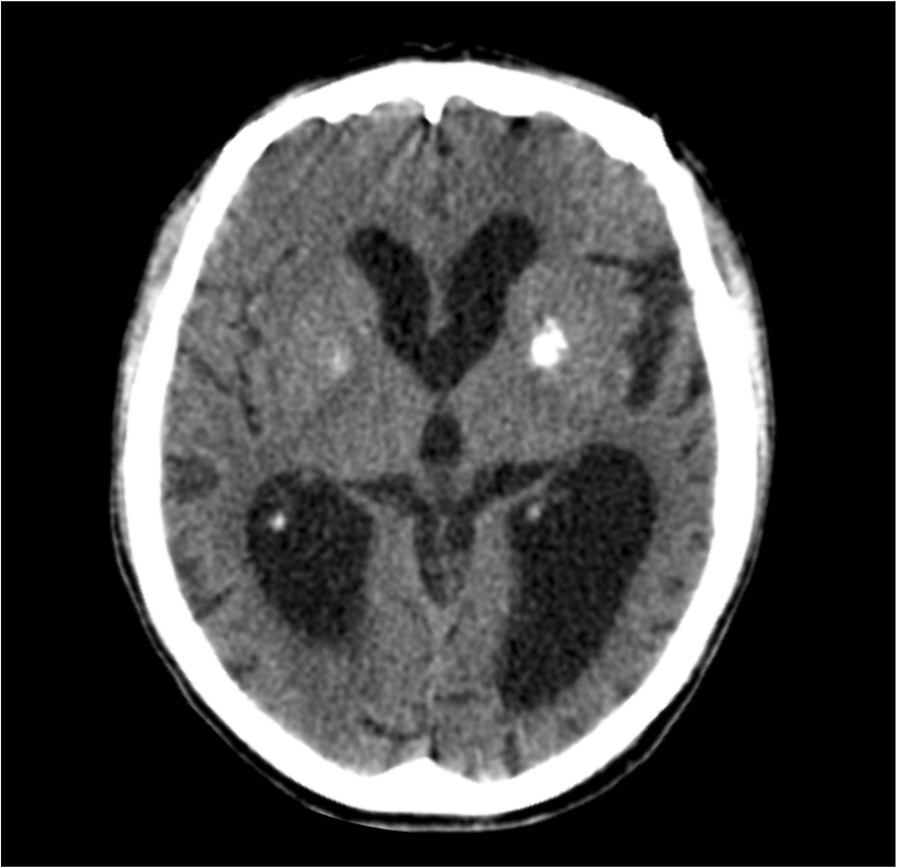
Axial CT of a 46-year-old Down syndrome patient with basal ganglia calcifications, mainly involving the *globus pallidus*

DS patients have a higher described frequency of intracranial calcifications (11–27%) [34, 35], and this frequency appears to increase with age. Nevertheless, the etiology and pathogenesis of this phenomenon is still not completely understood [34, 35]. On neuroimaging studies, brain calcifications in this syndrome are seen predominantly in the basal ganglia, especially the *globus pallidus* (Fig. 12).

### Malformations of the corpus callosum

Total or partial agenesis of the corpus callosum is one of the most common CNS malformations. It can result from prenatal infections, toxic exposure, vascular insults, and also from various genetic mutations and syndromes.

The association between malformations of the corpus callosum and DS has been occasionally reported in the literature, usually involving partial agenesis of the posterior segments of this structure [36–38].

Interestingly, one study of the structural chromosome rearrangements of individuals with anomalies of the corpus callosum identified a critical region on chromosome 21 [37], but more studies are needed to corroborate this finding.

## Spine and craniocervical junction

The prevalence of craniocervical instability in DS patients ranges between 8 and 63% [39]. The vast majority of cases is asymptomatic, with a very low prevalence of symptomatic cases (1% to 2%) [3, 40]. The ligament fragility has a foremost impact in these abnormalities; nonetheless, associated malformations in the bones of the craniovertebral junction are also contributing. Plain radiographs enable the assessment of the instability of the cervical spine, obtained in neutral positions and during flexion and extension; CT and MRI studies can also contribute to this evaluation.

**Fig. 13:**
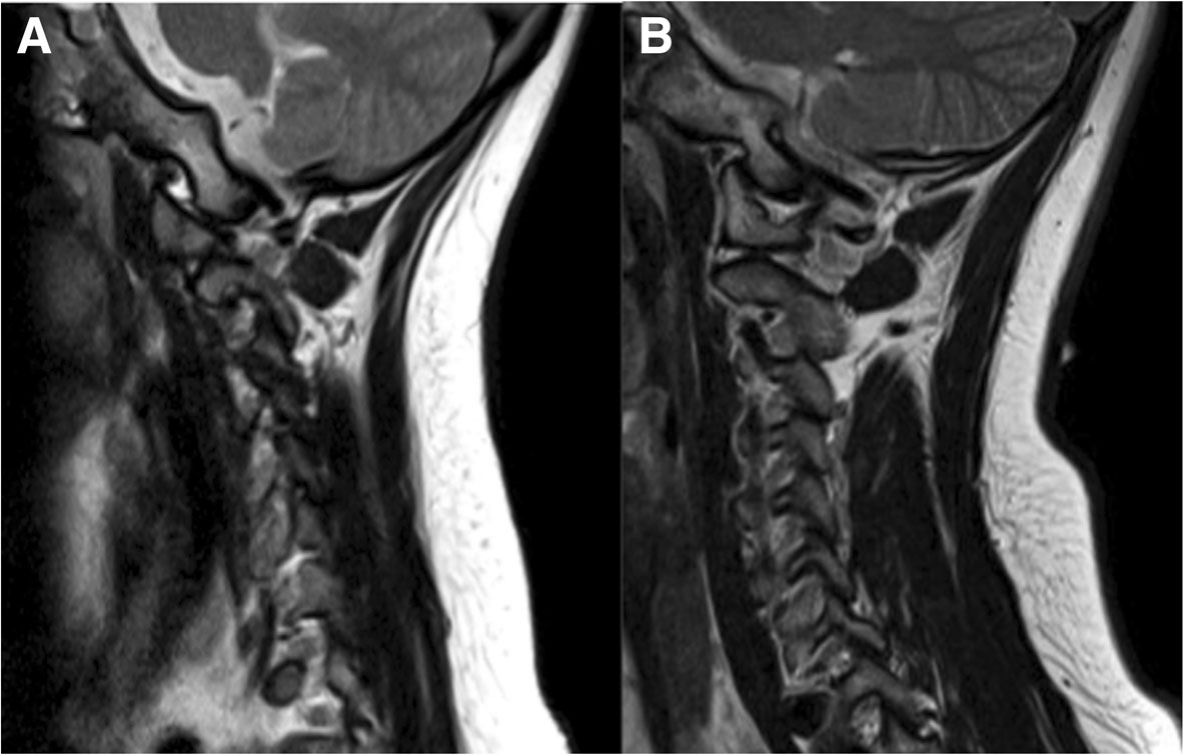
**a** Sagittal T2/TSE of a 12-year-old Down syndrome patient showing a small and flattened shape of the occipital condyle as well as the C1 superior articular facet, with some degree of anterior luxation of C1. **b** Sagittal T2/TSE of a normal 12-year-old boy for comparison; there is normal curved shape of articular facets

Concerning the occipito-cervical junction, some authors suggest that DS patients with congenital occipito-cervical instability do not develop the normal curved surfaces of the occipital condyles and the C1 superior articular facets, which become somewhat flattened [39, 40] (Fig. 13). This abnormality results in the deficiency of bony restraints to the exaggerated anterior and lateral translation, with ultimate failure in stability. As described earlier, ligamentous fragility can also add to this instability. Other common bony anomalies of the occipito-cervical region reported in DS patients are bifid anterior and/or posterior atlantal arches and atlanto-occipital assimilation [39].

**Fig. 14:**
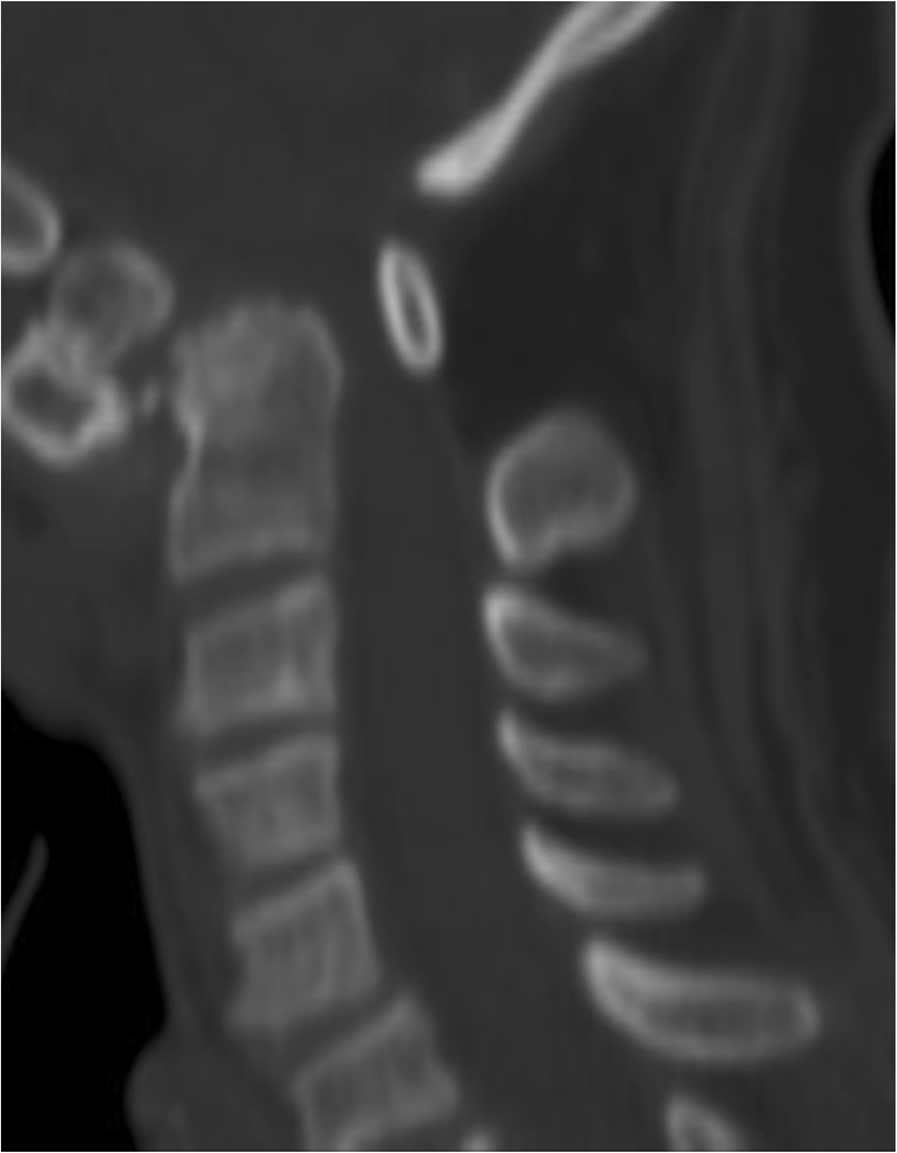
Sagittal cervical CT of a 68-year-old Down syndrome patient, showing the presence of an *os odontoideum* with atlanto-axial and atlanto-odontoid luxation; there is severe stenosis of the craniocervical junction, with space conflict in the bulbo-medullary junction

Regarding the atlanto-axial articulation in DS patients, instability can be secondary either to bony anomalies or to ligamentous defects and is seen in 10 to 30% of cases. The presence of congenital *os odontoideum* is reported in about 6% of DS children [39, 41] (Fig. 14). In this anomaly, the dens is not adequately fixed against the C1 anterior arch, leading to canal stenosis with flexion and extension movements; rotatory instability can also exist [39]. Other reported causes of atlanto-axial instability in DS patients are collagen defects leading to transverse ligament laxity [3, 39].

Surgical intervention in children with craniocervical instability is somewhat controversial [3, 39]. Generally, surgical fixation is considered when the patient has symptoms, when there is spinal cord compression or if the atlanto-occipital subluxation is superior to 7 mm [3, 39, 42]. Routine screening is moreover debatable. The American Academy of Pediatrics does not recommend screening; they advocate that plain radiographs cannot distinguish children with augmented risk of spinal complications. These guidelines recommend screening only in symptomatic individuals [43].

**Fig. 15:**
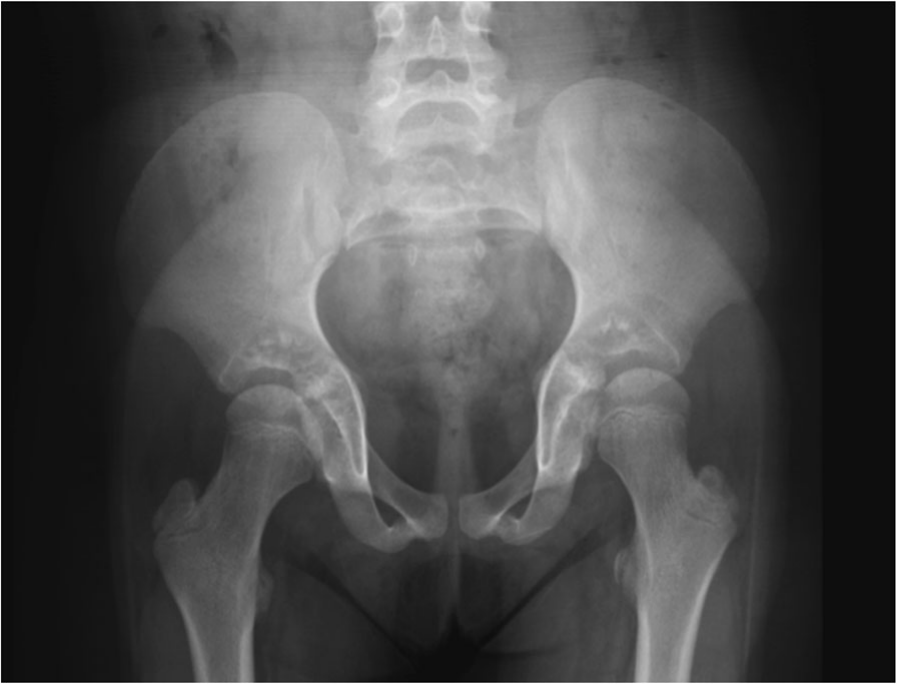
Plain radiograph of the pelvis in a 7-year-old Down syndrome girl, showing the typical “Mickey Mouse” pelvis

Spinal studies in DS patients frequently include the pelvis, which typically presents a “Mickey Mouse” morphology, with flaring of the iliac wings, a flat acetabular roof, and a small acetabular angle [3] (Fig. 15).

## Vascular abnormalities

### Moyamoya syndrome

Moyamoya syndrome is a rare arteriopathy characterized by progressive stenosis involving the apices of the intracranial internal carotid arteries (ICAs), as well as the proximal anterior and middle cerebral arteries. In response to this decrease in arterial flow into the brain, a network of collateral vessels usually develops. On cerebral angiography, these collateral vessels look like a “puff of smoke”, which stands for “moyamoya” in Japanese, hence the name to this syndrome [44].

The angiographic anomalies seen in moyamoya portrait a final pathway common to various congenital and acquired diseases. As such, the term “moyamoya syndrome” instead of “moyamoya disease” is applied whenever the patient has a well-recognized baseline condition, such as DS. Regarding moyamoya syndrome, almost 4% have DS and, of these, about 10% are under 15 years old [45]. Moyamoya syndrome is also seen in association with neurofibromatosis type 1, sickle cell anemia, as well as autoimmune disorders, and previous radiotherapy in the head and neck [44].

Patients with either moyamoya disease or syndrome can present with signs and symptoms resultant from ischemic injuries, from the insufficient blood supply, and/or from hemorrhagic complications from the fragile collateral vessels. It is reported that the majority of adults and children have ischemic injuries, although the frequency of hemorrhage is around seven times higher in adult patients (20% versus 3%) [44]. For children with moyamoya, it is extremely rare to have a hemorrhagic presentation; they usually experience transient ischemic attacks, acute ischemic strokes (68%) [46], and also headaches [44].

**Fig. 16:**
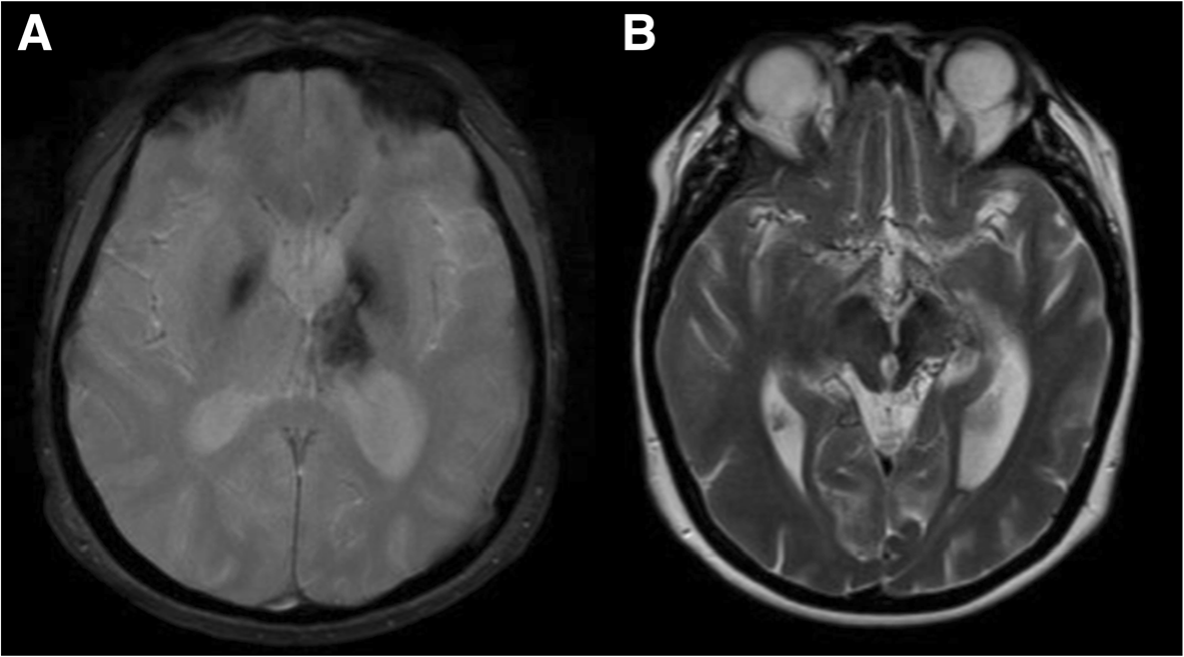
Axial T2* (**a**) and T2/TSE (**b**) of a 46-year-old patient with Down syndrome, presenting signs of an old hemorrhagic stroke involving the left thalamic-capsular area. Note also the prominent flow voids from collateral vessels involving the basal cisterns and horizontal segments of the Sylvian fissures. These features suggest associated moyamoya syndrome, which was confirmed in angiographic studies (not shown)

**Fig. 17:**
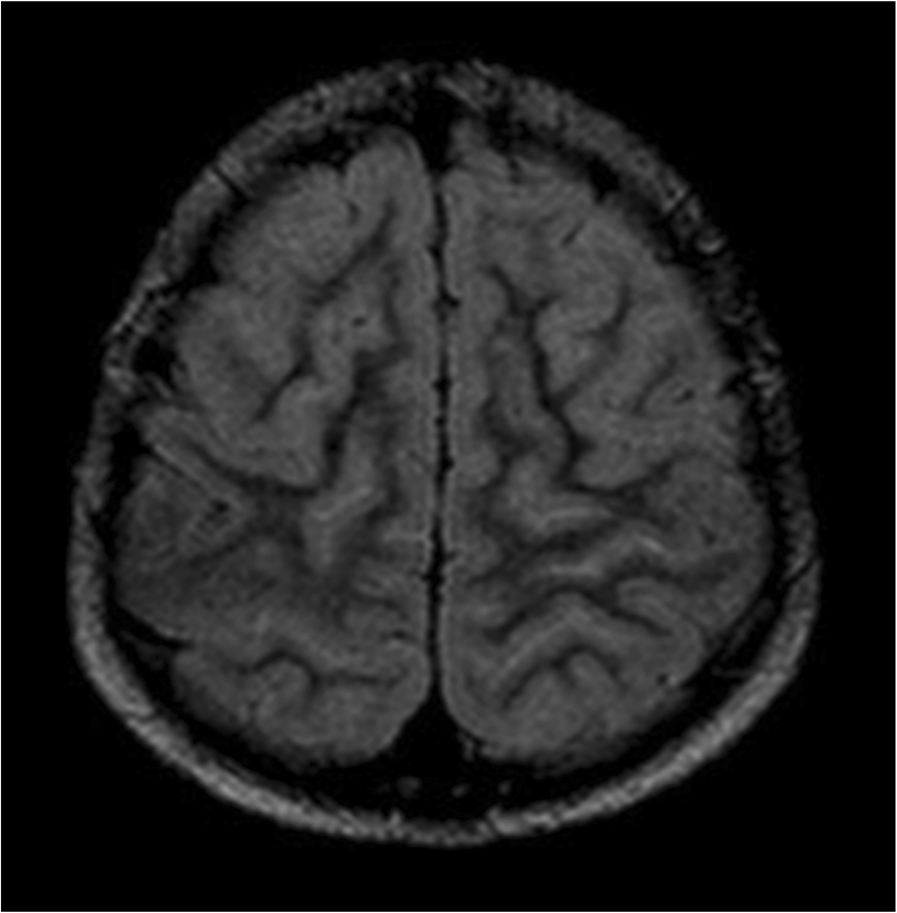
Axial FLAIR image in a 12-year-old girl with Down syndrome and moyamoya pattern depicting the classical *ivy sign*, corresponding to areas of absence of normal FLAIR suppression of CSF within cortical sulci. This sign probably represents slow flow in leptomeningeal collaterals. The exam was performed without general anesthesia

The imaging assessment of a patient suspected to have moyamoya syndrome includes CT and/or MRI of the brain with angiography and, in selected cases, also digital subtraction angiography (DSA). On CT, it is possible to identify hypodense areas corresponding to ischemic strokes, which are more common in watershed territories, the deep gray and white matter and periventricular zones [44]. Brain CT can also easily depict acute hemorrhages from the abnormal collaterals; the major sites of hemorrhage are the thalami, the basal ganglia, the ventricular system, and the medial temporal region. On MRI, acute and/or old ischemic or hemorrhagic strokes can usually be better identified (Fig. 16). Another typical imaging feature seen on MRI in moyamoya cases is the so-called *ivy sign*. It is represented by linear or curvilinear areas of leptomeningeal hyperintensity in fluid-attenuated inversion recovery (FLAIR) sequences (Fig. 17), probably related with slow flow in leptomeningeal collaterals. The lenticulostriate collaterals can also be seen in conventional MR sequences in moyamoya patients as numerous small and reticulated flow voids in the Sylvian fissures, thalami, and basal ganglia, especially on DP/T2 (Fig. 16).

**Fig. 18:**
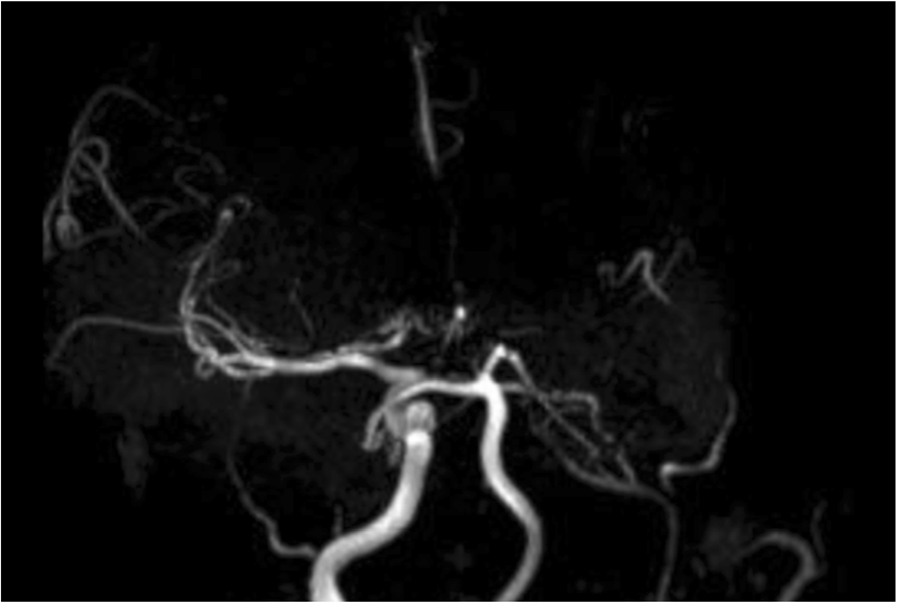
Three-dimensional reconstruction of MRI angiography (time-of-flight technique) of a 46-year-old Down syndrome patient, illustrating occlusion of the left internal carotid artery (ICA) and stenosis of the supraclinoid segment of the right ICA, as well as subocclusion of the right anterior cerebral artery (ACA). The left ACA and middle cerebral artery are not depicted. There are also multiple thin anomalous collateral vessels around the basal cisterns; these findings are compatible with moyamoya syndrome, which is known to have a high incidence in this population

**Fig. 19:**
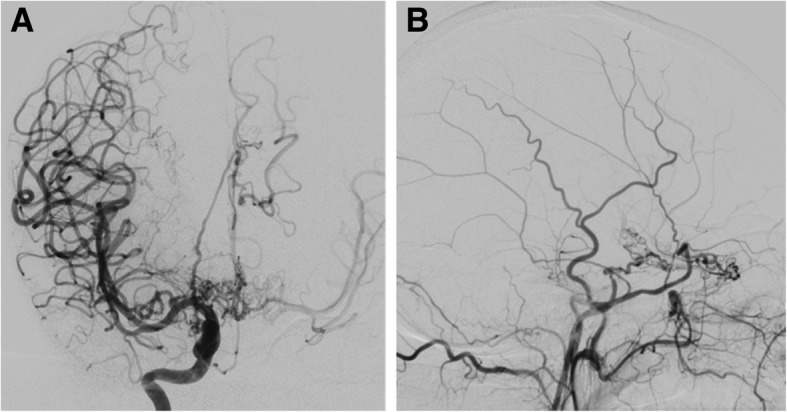
Cerebral angiography in a 46-year-old Down syndrome patient with moyamoya pattern. **a** Frontal view, right internal carotid artery injection, showing occlusion of the right anterior cerebral artery, with a network of collateral vessels (moyamoya vessels) in the supra-sellar cistern with reconstitution of the anterior cerebral artery arterial flow distally. **b** Lateral view, left internal carotid artery injection, showing severe narrowing of this artery, which ends on the cavernous segment, where the arterial flow diverges to a meningohypophyseal trunk with anastomosis with the ophthalmic artery and dural branches of the middle meningeal artery, forming a network of collateral capillaries, typical of moyamoya syndrome

Angiographic studies, including CT angiography (CTA) and MR angiography (MRA) (Fig. 18), demonstrate the typical features of this condition, namely narrowing/occlusion of the ICAs, middle and anterior cerebral arteries, and also the anomalous collateral vessels previously described. Cerebral DSA is still the diagnostic gold standard and is also valuable for characterization of the anatomy and flow of the vessels involved in the process (Fig. 19) [44], and it is performed specially when surgical treatment is considered.

Perfusion MR studies in moyamoya, including MR based cerebrovascular reactivity and hemodynamic reserve studies, are under intense research and can have a central role in the future clinical evaluation and management of these patients [47–52].

Concerning screening protocols for moyamoya syndrome in patients with DS and other predisposing related diseases, there is no class I data supporting this practice. However, in the particular case of DS patients, which is relatively common in pediatric practice, there is some class III data supporting prospective non-invasive screening for moyamoya syndrome [44]. Nevertheless, special attention should be paid to conventional brain MR sequences in all DS patients in order to identify features suggestive of this vascular disease as previously described and complement the exam with angiographic sequences whenever a suspicion is raised based on those images.

Treatment with cerebral revascularization in DS patients is only recommended in carefully selected patients [51]. Current therapies are not able to treat the primary disease process; instead, they act by preventing the occurrence of ischemic injuries, working in the improvement of blood flow to the non-healthy cerebral hemisphere. The use of surgical revascularization is regarded as the primary treatment option in the literature, including the last guidelines from the American Heart Association [52]. Various surgical techniques have been described, all aiming to prevent additional ischemic injury by improving the collateral blood flow, usually by using the external carotid circulation [44].

### Aberrant subclavian artery

Aberrant right subclavian artery (ARSA), also known as *arteria lusoria*, is a rare vascular condition where the right subclavian artery instead of being the first branch of the aortic arch (with right common carotid artery forming the brachiocephalic trunk) arises isolated as the fourth branch, after the origin of the left subclavian artery, and then turns back to reach the right side.

In recent literature, it has been proposed that the prenatal identification of an ARSA is markedly enlarged in DS fetus, with a prevalence of 19 to 36% [53]. This can be a useful ultrasonographic marker of DS in first trimester screenings, although still not routinely performed. In addition, fetal karyotype testing when this finding is present as an isolated finding is still a debatable issue [54]; nevertheless, it is recommended if there is a background risk or if there are additional markers present [55].

**Fig. 20:**
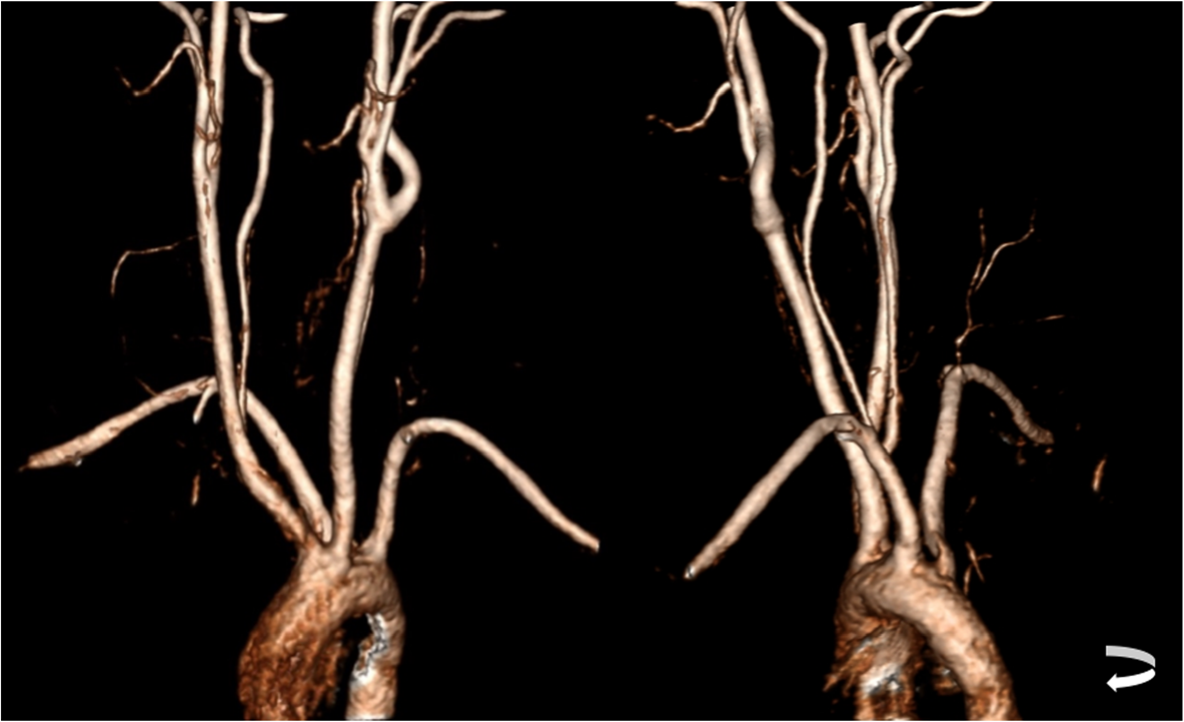
Three-dimensional reconstruction of a CT angiography of the supra-aortic trunks of a 49-year-old patient with Down syndrome depicting an aberrant right subclavian artery

ARSA can be easily showed in angiographic studies, namely MRA and CTA (Fig. 20). This normal variant should be assessed and reported because it may lead to feeding problems in DS patients due to compression of the esophagus by the abnormal artery. However, the existence of an ARSA does not necessarily lead to feeding difficulties, and surgical treatment should only be considered in carefully chosen patients [53].

### Future directions in neuroimaging in DS

The combination of several neurological features in DS patients, such as language impairment, cognition, learning, and memory, has given rise to intense neurodevelopmental research in these patients. Indeed, besides volumetric changes, there are also probably structural and functional brain abnormalities in DS patients which may be further characterized by advanced neuroimaging techniques.

MR spectroscopy studies in DS patients with or without dementia have shown an elevated peak of myoinositol, but no abnormalities in the creatine or *N*-acetylaspartate [9]. On the other side, task-related functional MRI (fMRI) have shown reduced activation in language areas and distinctive relations between activation and visuo-spatial capability in DS patients compared with controls [56–58].

Recently, there has been emergent interest in potential modifications in anatomical connectivity in this syndrome. Functional MRI connectivity (fcMRI) is based on the premises that functionally associated brain regions have temporally synchronized oscillations on blood oxygen level dependent (BOLD) signal [58]. Therefore, by assessing the temporal connection amongst brain regions, fcMRI can schematize a functional network anatomy. A study published in the literature concerning brain connectivity in DS patients reported a widespread increased synchrony between brain regions, with a temporal pattern, which was not related to environmental stimuli; instead, it was distinctive to a single DS patient. This can result from poor or non-existent negative links between different brain areas and also a reduction of long-distance connections. These findings suggest an immature brain connectivity development in DS patients, along with compromised ability to incorporate information that originates in distant brain areas into logically organized networks [9].

Diffusion tensor imaging (DTI) is a non-invasive MRI study used to characterize the structure of the white matter tracts in vivo, by quantitatively determining the diffusion of water molecules, and is becoming a widespread instrument for assessment of structural connectivity. There is a paucity of published studies using DTI in the evaluation of DS patients. One published study reported a decline on the fractional anisotropy (FA) in DS adult patients when compared to controls, with an association to poorer cognition [59]. Even rarer studies are published concerning DTI in children with DS. One study reported similar findings in children between 2 and 4 years old [10].

In the future, the use of these and other new advanced MR techniques in larger groups of DS patients may contribute to provide a wider understanding about the brain tissue microstructure and function in DS and what lies beneath the neurodevelopmental and neurodegenerative process in these particular patients.

## Abbreviations

AD: Alzheimer disease
ARSA: Aberrant right subclavian artery
BPD: Biparietal diameter
CHL: Conductive hearing loss
CT: Computed tomography
CTA: CT angiography
DS: Down syndrome
DSA: Digital subtraction angiography
DTI: Diffusion tensor imaging
FA: Fractional anisotropy
fcMRI: Functional MRI connectivity
FLAIR: Fluid-attenuated inversion recovery
fMRI: Functional MRI
IAC: Internal auditory canal
ICA: Internal carotid artery
LOMEDS: Late-onset myoclonic epilepsy in Down syndrome
LSCC: Lateral semi-circular canal
MRA: MR angiography
MRI: Magnetic resonance imaging
OFD: Occipitofrontal diameter
SNHL: Sensorineural hearing loss
